# Descending colon fistula: Unusual complication of severe acute pancreatitis a case report

**DOI:** 10.1016/j.amsu.2022.103426

**Published:** 2022-03-01

**Authors:** Haithem Zaafouri, Atif Dawood, Meriam Mesbahi, Turki Alotaibi, Mourouj A.L. Ahmadi, Maged Aiat

**Affiliations:** a Department of General Surgery, King Abdul Aziz Hospital, Jeddah, Saudi Arabia; b General Surgery Department, Habib Thameur Hospital, Tunis, Tunisia

**Keywords:** Acute pancreatitis, Colonic fistula, Drainage, Conservative management, Case report

## Abstract

**Introduction:**

The incidence of colonic complications from acute pancreatitis (AP) and severe AP are 3.3% and 15%, respectively. We report a case of descending colon fistula secondary to severe AP and its management.

**Case presentation:**

We report a case of a 35-year-old male hospitalized in our department for severe acute pancreatitis (grade E of Balthazar classification).

Initially, the evolution was favorable under medical management. Two months later, he was readmitted for infection of the necrosis with a descending colon fistula. As we did not have the possibility of performing a CT scan drainage, our plan was to do surgical drainage under general anesthesia.

**Conclusion:**

The colonic involvement following AP or severe AP is rare and difficult to diagnoses. Conservative treatment when some conditions are available should be the best choice; it is associated with lower risk of morbidity and mortality.

## Highlights

• Knowledge about colonic complications from AP has been limited to few case reports.
• Thus diagnostic and management dilemmas continue to persist. The clinical presentation can be variable.
• Nonspecific and could occur quite late in the disease process. For the management.
• There are no evidence-based guidelines.

## 1 Introduction

Acute pancreatitis (AP) is associated with diverse complications. Gastrointestinal complications are rare [[1], [2], [3], [4]] like hemorrhage, ischemic colitis, necrosis, fistula and perforation. The incidence of colonic complications from AP and severe AP are 3.3% and 15%, respectively [4].

We report a case of descending colon fistula secondary to severe AP and its management. This work has been reported concerning the SCARE 2020 criteria [5].

## 2 Case presentation

A 35-year-old male patient, without past medical history, presented to the emergency department with an acute epigastric pain without radiation and vomiting. He denied any allergies neither drug history. He reported having a high fat meal the preceding evening. He complained of abdominal distention and that he don't passing stools or gas for 24 hours.

On admission, He had stable vital signs and abdominal examination revealed tenderness in the epigastrium. The admission workup noted for an amylase level of 4727, a lactate level of 2.2 and white blood cell count of 19.

**Fig. 1:**
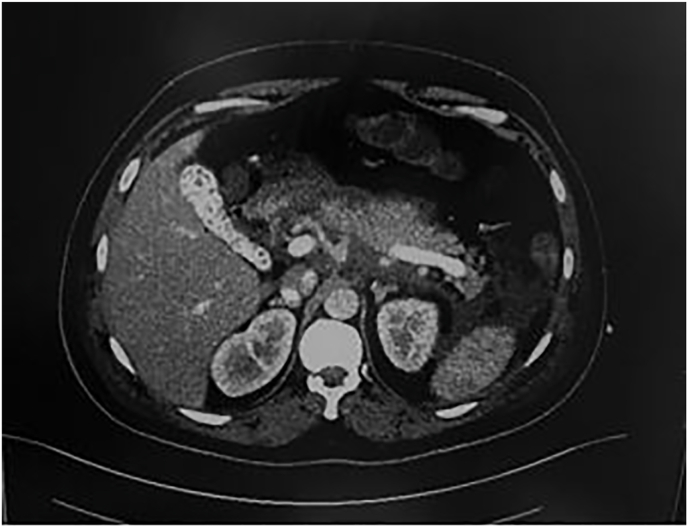
**The computed tomography findings** showed severe AP with extensive inflammatory stranding around the pancreas, free fluid in the left paracolic gutters (grade E of Balthazar classification), and a gallbladder stone with no evidence of biliary dilatation or free air.

The computed tomography of the abdomen and pelvis showed an evidence of severe AP with extensive inflammatory stranding around the pancreas, free fluid in the left paracolic gutters (grade E of Balthazar classification), and a gallbladder stone with no evidence of biliary dilatation or free air (Fig. 1).

The diagnosis of biliary severe acute pancreatitis was retained. Our management was to keep the patient on NPO for 48 hours with an infusion, analgesics, omeprazole and monitoring of hemodynamic and biological constants.

The evolution was favorable, clinically the patient tolerated oral feeding and the results of the biological assessment were normal.

A CT scan planned but the patient refused the examination and left the hospital against medical advice.

Two months later, he was readmitted through the emergency department for abdominal pain.

On clinical examination, he was afebrile; the abdomen was flexible and depressible with tenderness of the left flank. In biology there was no hyperleukocytosis and amylasemia was normal.

**Fig. 2:**
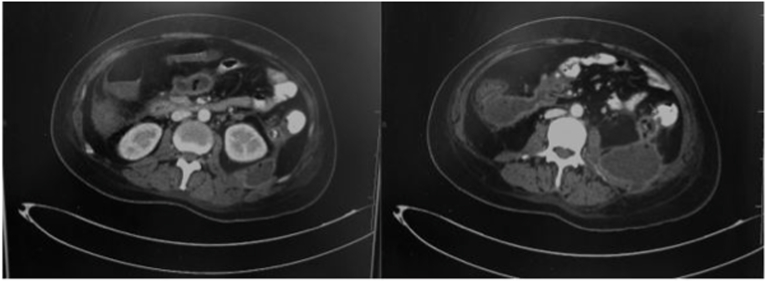
A+2B: **The abdominal CT scan** showed an extensive necrosis with the presence of air bubbles, and the existence of a fistula of the descending colon in the left retroperitoneal space.

An abdominal CT scan concluded that the necrosis flows had extended up to the pelvis with the presence of air bubbles testifying to the infection. The CT also showed the existence of a fistula of the descending colon in the left retroperitoneal space (Fig. 2A and B).

As we did not have the possibility of performing a CT scan drainage, our plan was to do surgical drainage under general anesthesia.

The first step was to perform a laparoscopic exploration of the abdominal cavity to exclude intraperioneal communication of the colonic fistula and to verify the presence of a collection secondary to the infection of the necrosis flows.

The second step was to explore the left retro peritoneal cavity, the descending colon was of good vitality and the colonic fistula was clogged. A washing of the cavity and installation of a gauge drain were done.

The follow up was favorable and the drain was removed on day 9 postoperatively. The patient reported being satisfied with the intervention, and the postoperative course was uneventful. The patient remains asymptomatic.

## 3 Discussion

Knowledge about colonic complications from AP has been limited to few case reports, thus diagnostic and management dilemmas continue to persist.

Colonic complications in AP are uncommon and include necrosis, obstruction, fistula formation, and perforation.

Generally, the transverse colon is involved with complications related to AP [6]. Little has been reported on descending colon pathology in association with AP: case of stenosis after severe AP [7] and case of stercoral perforation [8]**.** Recently, Dhadlie [9] report one case of ascending colon perforation.

Several theories have been proposed to explain the involvement of colon in AP:

• Ischemia: peri pancreatic inflammation can affect the vascular watershed area of the colon around the splenic flexure witch it is closely abutting the tail of the pancreas. This is the main reason to explain that splenic flexure is the most common site of colon involvement. Hypotension from severe pancreatitis aggravates the predisposition to ischemia at this watershed area [10].
• Thrombosis: edema in transverse mesocolon with hypotension causes mesenteric and submucosal vessels thrombosis and can precipitate colon necrosis [10].

This both theories caused intestinal complications by indirect damage. Also there is one theory to explain colonic involvement by direct damage proposed by Minnen [11]:

• Direct damage: propagation of retroperitoneal spread of pancreatic enzymes and inflammatory substances to the mesocolon causing pericolitis, transmural necrosis, and perforation.
• Pseudocyst formation has also been commonly suggested as the mechanism.

The median period until the occurrence of necrosis, irrespective of perforation, was reported as 25 days [4]. Nakanishi [12] in his study found that the minimum interval between AP onset and development of colonic perforation was 6 days, with a median interval of 13 days.

Clinical symptoms, blood test data, and computed tomography (CT) findings seen at AP onset were examined as risk factors of colonic perforation [12]. Univariate analyses identified Balthazar grade E as a risk factor (*p* = 0.0087) [12]. This finding was consistent with almost all previous studies, which also found that intestinal complications occur at Balthazar grade D or greater [[13], [14], [15], [16]].

A relationship between colonic perforation, alcoholic pancreatitis and presence of pseudocyst was also found with marginal significance (p = 0.06 and 0.053 respectively) [17,18].

The clinical presentation can be variable, non specific and could occur quite late in the disease process [4].

For the management, there are no evidence-based guidelines because such cases mainly have been reported as case reports and series.

There are two options for the management of the colonic involvement:

• Conservative measures such as drainage, IV antibiotic and parenteral nutrition during fifteen to twenty-one days.
• Surgical intervention with colon resection.

The clinical presentation of the patient as well as the presence of sepsis or septic shock, the site of the colonic involvement and especially if the fistula or the perforation is in the retro peritoneal or intra peritoneal side.

Although surgical intervention with colon resection may be difficult, complicated and associated with higher risk of mortality, it should be reserved only for nonviable colon. In Aldridge series [9], ten patients had colon resection and six of them resection may be not necessary (histopathological examination showed only pericolitis).

## 4 Conclusion

The colonic involvement following AP or severe AP is rare and difficult to diagnoses. The physician needs to be aware of it, and any delay in its management can increase morbidity and mortality. Conservative treatment when some conditions are available should be the best choice; it is associated with lower risk of morbidity and mortality.

## Compliance with ethical standards

The patient has provided both verbal and written consent for the publication of This article. It was made sure that his identity will be kept a secret at all levels.

## Funding

None.

## Author contributions

All authors were involved in the researching, writing, and editing of the manuscript.

## Conflict of interest for all authors

The authors declare no competing interest.

## Research registration

Not applicable.

## Guarantor

Haithem Zaafouri.

## Consent

Written informed consent was obtained from the patient for publication of this case report and accompanying images. A copy of the written consent is available for review by the Editor-in-Chief of this journal on request.

## Provenance and peer review

Not commissioned, externally peer-reviewed.
